# Supplementary material for: Pbp1, the yeast ortholog of human Ataxin-2, functions in the cell growth on non-fermentable carbon sources
Source: PLoS One. 2021 May 13;16(5):e0251456. doi: 10.1371/journal.pone.0251456 (PMC8118320; doi:10.1371/journal.pone.0251456)
Supplement: S1 Table — (DOCX) [file pone.0251456.s001.docx]

**S1 Table. Strains used in this study.**

| **Strains** | **Genotype** | **Reference** |
| --- | --- | --- |
| 10B | *MATα ade2 trp1 can1 leu2 his3 ura3 GAL psi+ HOp-ADE2-HO 3' UTR* | 51 |
| 10BD | *MATa/MATα ade2/ade2 trp1/trp1 can1/can1 leu2/leu2 his3/his3 ura3/ura3* | 51 |
| *pbp1∆-1* | *MATa ade2 trp1 can1 leu2 his3 ura3 pbp1Δ::CgLEU2* | This study |
| *pbp1∆-2* | *MATa ade2 trp1 can1 leu2 his3 ura3 pbp1Δ::CgHIS3* | This study |
| *tor1∆* | *MATa ade2 trp1 can1 leu2 his3 ura3 tor1Δ::CgHIS3* | This study |
| *pbp1∆ tor1∆* | *MATa ade2 trp1 can1 leu2 his3 ura3 pbp1Δ::CgLEU2 tor1Δ::CgHIS3* | This study |
| *pan2∆* | *MATa ade2 trp1 can1 leu2 his3 ura3 pan2Δ::CgLEU2* | This study |
| *pbp1∆ pan2∆* | *MATa ade2 trp1 can1 leu2 his3 ura3 pan2Δ::CgLEU2 pbp1Δ::CgHIS3* | This study |
| *ccr4∆* | *MATa ade2 trp1 can1 leu2 his3 ura3 ccr4Δ::CgLEU2* | This study |
| *pbp1∆ ccr4∆* | *MATa ade2 trp1 can1 leu2 his3 ura3 ccr4Δ::CgLEU2 pbp1Δ::CgHIS3* | This study |
| *cox10∆* | *MATa ade2 trp1 can1 leu2 his3 ura3 cox10Δ::CgLEU2* | This study |
| *pbp1∆ cox10∆* | *MATa ade2 trp1 can1 leu2 his3 ura3 cox10Δ::CgLEU2 pbp1Δ::CgHIS3* | This study |
| *cat8∆* | *MATa ade2 trp1 can1 leu2 his3 ura3 cat8Δ::CgLEU2* | This study |
| *pbp1∆ cat8∆* | *MATa ade2 trp1 can1 leu2 his3 ura3 pbp1Δ::CgHIS3 cat8Δ::CgLEU2* | This study |
| *xrn1∆* | *MATa ade2 trp1 can1 leu2 his3 ura3 xrn1Δ::CgLEU2* | This study |
| *pbp1∆ xrn1∆* | *MATa ade2 trp1 can1 leu2 his3 ura3 pbp1Δ::CgHIS3 xrn1Δ::CgLEU2* | This study |
| *dhh1∆* | *MATa ade2 trp1 can1 leu2 his3 ura3 dhh1Δ::CgLEU2* | This study |
| *pbp1∆ dhh1∆* | *MATa ade2 trp1 can1 leu2 his3 ura3 pbp1Δ::CgHIS3 dhh1Δ::CgLEU2* | This study |
| *Dcp1-AID* | *MATa ade2 trp1 can1 leu2 his3 ura3 DCP1-AIDkanMX6* | This study |
| *Dcp1-AID [TIR1]* | *MATa ade2 trp1 can1 leu2 his3 ura3 DCP1-AIDkanMX6 [TIR1]* | This study |
| *pbp1∆ Dcp1-AID [TIR1]* | *MATa ade2 trp1 can1 leu2 his3 ura3 pbp1Δ::CgHIS3 DCP1-AID::kanMX6 [TIR1]* | This study |

Reference

51. Tadauchi T, Matsumoto K, Herskowitz I, Irie K. Post-transcriptional regulation through the HO 3'-UTR by Mpt5, a yeast homolog of Pumilio and FBF. EMBO J. 2001 Feb 1;20(3):552-61. doi: 10.1093/emboj/20.3.552.
