# Supplementary material for: Pbp1, the yeast ortholog of human Ataxin-2, functions in the cell growth on non-fermentable carbon sources
Source: PLoS One. 2021 May 13;16(5):e0251456. doi: 10.1371/journal.pone.0251456 (PMC8118320; doi:10.1371/journal.pone.0251456)
Supplement: S2 Table — (DOCX) [file pone.0251456.s002.docx]

**S2 Table. Plasmids used in this study.**

| **Plasmids** | **Relevant markers** | **Reference** |
| --- | --- | --- |
| pCgLEU2 | *C. glabrata LEU2* in pUC19 | 29 |
| pCgHIS3 | *C. glabrata HIS3* in pUC19 | 29 |
| pCgTRP1 | *C. glabrata TRP1* in pUC19 | 29 |
| pFA6A-GFP(S65T)-KanMX6 | *GFP-ADH1* terminator*,* kanamycin resistance cassette | 27 |
| pFA6a-3HA-kanMX6 | *3HA-ADH1* terminator, kanamycin resistance cassette | 27 |
| YCplac33 | *URA3, CEN-ARS* | 52 |
| YCplac33-*pPCK1*-*GFP* | *URA3, CEN-ARS, pPCK1-GFP-ADH1* terminator | This study |
| YCplac33-*pFBP1*-*GFP* | *URA3, CEN-ARS, pFBP1-GFP-ADH1* terminator | This study |
| YCplac33-*pICL1*-*GFP* | *URA3, CEN-ARS, pICL1-GFP-ADH1* terminator | This study |
| YCplac33-*pCOX10*-*GFP* | *URA3, CEN-ARS, pCOX10-GFP-ADH1* terminator | This study |
| YCplac33-*pCOX11*-*GFP* | *URA3, CEN-ARS, pCOX11-GFP-ADH1* terminator | This study |
| YCplac33-*pCYT2*-*GFP* | *URA3, CEN-ARS, pCYT2-GFP-ADH1* terminator | This study |
| YCplac33-*pMRPL3*-*GFP* | *URA3, CEN-ARS, pMRPL3-GFP-ADH1* terminator | This study |
| YCplac33-*pMRPS35*-*GFP* | *URA3, CEN-ARS, pMRPS35-GFP-ADH1* terminator | This study |
| YCplac33-*pMSY1-GFP* | *URA3, CEN-ARS, pMSY1-GFP-ADH1* terminator | This study |
| YCplac33-*pAIM33*-*GFP* | *URA3, CEN-ARS, pAIM33-GFP-ADH1* terminator | This study |
| YCplac33-*pIBA57*-*GFP* | *URA3, CEN-ARS, pIBA57-GFP-ADH1* terminator | This study |
| YCplac33-*pCOX10*-*GFP-COX10 3’-UTR* | *URA3, CEN-ARS, pCOX10-GFP-COX10 3’-UTR* | This study |
| YCplac33-*pCOX11*-*GFP- COX11 3’-UTR* | *URA3, CEN-ARS, pCOX11-GFP-COX11 3’-UTR* | This study |
| YCplac33-*COX10* | *URA3, 2µ, pCOX10-COX10-COX10 3'-UTR* | This study |
| pMK43 | C-terminal *IAA17* tag, kanamycin | 39 |
| pMK76 | *ADH1* promoter-*AtTIR1-9Myc*, *URA3* | 39 |

Reference

52. Gietz R D, Sugino A. New yeast-Escherichia coli shuttle vectors constructed with in vitro mutagenized yeast genes lacking six-base pair restriction sites. Gene. 1988 Dec 30;74(2):527-34. doi: 10.1016/0378-1119(88)90185-0.
