# Supplementary material for: Pbp1, the yeast ortholog of human Ataxin-2, functions in the cell growth on non-fermentable carbon sources
Source: PLoS One. 2021 May 13;16(5):e0251456. doi: 10.1371/journal.pone.0251456 (PMC8118320; doi:10.1371/journal.pone.0251456)
Supplement: S3 Table — (DOCX) [file pone.0251456.s003.docx]

**S3 Table. Primers used in this study**

| **Primers** | **Sequence 5’-3’** | **Purpose** |
| --- | --- | --- |
| tv1*PCK1*pro-33-F | CAAGCTTGCATGCCTGCAGGTCGACCACAGTCGACGAGTTTGTCT | *PCK1*pro-GFP |
| tv2*PCK1*pro-GFP-R | GTTAATTAACCCGGGGATCCGGGACATGTTGTTATTTTATTATGG |  |
| tv3*GFP*-*PCK1*pro-F | CCATAATAAAATAACAACATGTCCCGGATCCCCGGGTTAATTAAC |  |
| tv4*ADH1*-33-R | TTGTAAAACGACGGCCAGTGAATTCATCTATATTACCCTGTTATCC |  |
| tv5*COX10*pro-33-F | CAAGCTTGCATGCCTGCAGGTCGACACCGACGAATACGGCGGAAC | *COX10*pro-GFP |
| tv6*COX10*pro-GFP-R | GTTAATTAACCCGGGGATCCGACTCATCTAAAGAGGAAAAAAGGG |  |
| tv7*GFP-COX10pro*-F | CTCCTTTTTTCCTCTTTAGATGAGTCGGATCCCCGGGTTAATTAAC |  |
| tv8*ICL1*pro-33-F | CAAGCTTGCATGCCTGCAGGTCGACTGGAAATGTAAAGGATAATG | *ICL1*pro-GFP |
| tv9*ICL1*pro-*GFP*-R | GTTAATTAACCCGGGGATCCGAGGCATTTTTCGTTGACTTTTTGT |  |
| tv10*GFP*-*ICL1*pro-F | ACAAAAAGTCAACGAAAAATGCCTCGGATCCCCGGGTTAATTAAC |  |
| tv11*FBP1*pro-33-F | CAAGCTTGCATGCCTGCAGGTCGACGCCAAGGAAGGTGGGTTTAC | *FBP1*pro-GFP |
| tv12*FBP1*pro-*GFP*-R | GTTAATTAACCCGGGGATCCGTGGCATATGTGTGGTAGTATGAGGG |  |
| tv13*GFP*-*FBP1*pro-F | CCCTCATACTACCACACATATGCCACGGATCCCCGGGTTAATTAA |  |
| tv14*COX11*pro-33-F | CAAGCTTGCATGCCTGCAGGTCGACTTGTGTGGTTTGTGAGCCATTTTC | *COX11*pro-GFP |
| tv15*COX11*pro-GFP-R | GTTAATTAACCCGGGGATCCGGGACATATTCTTATCATGACAATA |  |
| tv16*GFP*-*COX11*pro-F | TATTGTCATGATAAGAATATGTCCCGGATCCCCGGGTTAATTAA |  |
| tv17*CYT2*pro-33-F | CAAGCTTGCATGCCTGCAGGTCGACAATGCCGAGAAGGGGGGGAGAA | *CYT2*pro-GFP |
| tv18*CYT2*pro-*GFP*-R | GTTAATTAACCCGGGGATCCGCATCATAATATGTAAAATAGAGACG |  |
| tv19*GFP*-*CYT2*pro-F | CGTCTCTATTTTACATATTATGATGCGGATCCCCGGGTTAATTAA |  |
| tv20*MRPL3*pro-33-F | CAAGCTTGCATGCCTGCAGGTCGACAGGATCTCGAAATAAGCCGTG | *MRPL3*pro-GFP |
| tv21*MRPL3*pro-GFP-R | GTTAATTAACCCGGGGATCCGGCCCATTCTGTATATATAACTACAGC |  |
| tv22*GFP*-*MRPL3*pro-F | GCTGTAGTTATATATACAGAATGGGCCGGATCCCCGGGTTAATTAA |  |
| tv23*MRPS35*pro-33-F | CAAGCTTGCATGCCTGCAGGTCGACCTGGAGAATATGCTAGATAATTTA | *MRPS35*pro-GFP |
| tv24*MRPS35*pro-*GFP*-R | GTTAATTAACCCGGGGATCCGACTCATCACAATCGAATATTACTA |  |
| tv25*GFP*-*MRPS35*pro-F | TAGTAATATTCGATTGTGATGAGTCGGATCCCCGGGTTAATTAA |  |
| tv26*MSY1*pro-33-F | CAAGCTTGCATGCCTGCAGGTCGACTGAAACAGCTCTTCGTTGTTG | *MSY1*pro-GFP |
| tv27*MSY1*pro-*GFP*-R | GTTAATTAACCCGGGGATCCGGAGCATGATTCACAATGTCTACCTG |  |
| tv28*GFP*-*MSY1*pro-F | CAGGTAGACATTGTGAATCATGCTCCGGATCCCCGGGTTAATTAA |  |
| tv29*AIM33*pro-33-F | CAAGCTTGCATGCCTGCAGGTCGACTCATTGGATCATTCTATTGCGC | *AIM33*pro-GFP |
| tv30*AIM33*pro-GFP-R | GTTAATTAACCCGGGGATCCGCGACATTGTTGCGAATGGTTGC |  |
| tv31*GFP*-*AIM33*pro-F | GCAACCATTCGCAACAATGTCGCGGATCCCCGGGTTAATTAA |  |
| tv32*IBA57*pro-33-F | CAAGCTTGCATGCCTGCAGGTCGACATTGGATGCCGCCGTTGTCG | *IBA57*pro-GFP |
| tv33*IBA57*pro-GFP-R | GTTAATTAACCCGGGGATCCGGAACATGTCCAGTGGGAAAGCGA |  |
| tv34*GFP*-*IBA57*pro-F | TCGCTTTCCCACTGGACATGTTCCGGATCCCCGGGTTAATTAA |  |
| tvt50*COX10*-F | GGATTGGATATATCCTGGTGAAGCAAAGCGACCACAGGAACGATTTACGGATCCCCGGGTTAATTAA | Switch *COX10* 3'UTR to *ADH1* ter in endogenous *COX10* gene |
| tvt51*COX10*-R | GACTGCCCTTTAAGCGTTGTCTCTTTATCTCATTGTACTAATGGAATTCGAGCTCGTTTAAAC |  |
| tvt52*COX11*-F | ATTTTATCCCCAGAAGTTATAGACACAAGGAAAGACAACTCAAATACGGATCCCCGGGTTAATTAA | Switch *COX11* 3'UTR to *ADH1* ter in endogenous *COX11* gene |
| tvt53*COX11*-R | GCATAACAGTATTTATGTACATTGAATGACTTTGTATTTACAAGAATTCGAGCTCGTTTAAAC |  |
| tvc54*COX10*-R | GTCTCTTTATCTCATTGTACTAATGTTATTTAGAAGTGGCGCGCCCTATT | Switch *ADH1* ter to *COX10* 3' UTR in *COX10*p-*GFP* reporter gene |
| tvc55*COX10*3'-F | AATAGGGCGCGCCACTTCTAAATAACATTAGTACAATGAGATAAAGAGAC |  |
| tvc56*COX10*3'-R | TTGTAAAACGACGGCCAGTGAATTCCTTGACAGCGAAAGATATAGCTAAG |  |
| tvc57*COX11*-R | ACATTGAATGACTTTGTATTTACAATTATTTAGAAGTGGCGCGCCCTATT | Switch *ADH1* ter to *COX11* 3' UTR in *COX11*p-*GFP* reporter gene |
| tvc58*COX11*3'-F | AATAGGGCGCGCCACTTCTAAATAATTGTAAATACAAAGTCATTCAATGT |  |
| tvc59*COX11*3'-R | TTGTAAAACGACGGCCAGTGAATTCTTGCTGAGATTTGTGACGGTGTCTT |  |
