# Supplementary material for: Pbp1, the yeast ortholog of human Ataxin-2, functions in the cell growth on non-fermentable carbon sources
Source: PLoS One. 2021 May 13;16(5):e0251456. doi: 10.1371/journal.pone.0251456 (PMC8118320; doi:10.1371/journal.pone.0251456)
Supplement: S4 Table — (DOCX) [file pone.0251456.s004.docx]

**S4 Table. Primers for RT-PCR used in this study**

| gene | forward primer | reverse primer |
| --- | --- | --- |
| *ACT1* | TGCCGAAAGAATGCAAAAGG | TCTGGAGGAGCAATGATCTTGA |
| *SCR1* | AACCGTCTTTCCTCCGTCGTAA | CTACCTTGCCGCACCAGACA |
| *PCK1* | GTTTGTGCCAGGGCTTACCA | GGAACTGACCAGCGTTCCAG |
| *FBP1* | CCCACAAACACGGGCTCATA | GACGCGATAGTTCCAACGGA |
| *ICL1* | GCCAAGAAGCTCGCTAAAGAAA | TAGAACATTGCGTCCCACCTC |
| *COX10* | GGTTAGGTGCCCTAGTAGGAATGG | AAGCAAACAATAGACCAGCAAGG |
| *COX11* | TGCGAGAGTTGAAAAGAGCAAG | CCAAAACCAGTACGAGCACAAA |
| *CYT2* | TGGTTCAGGTCCCGTATCTTG | TGGTTGTTGCTGCGAGTTTG |
| *PBP1* | TCCATCGGCATCACCAAA | CTTCTTCAGCCCCATTGCTAC |
| *GFP* | CACTGGAGTTGTCCCAATTCTTG | TCCGTATGTTGCATCACCTTCA |
| *AIM33* | ACGGTAAATGGAACGGTGATG | GGTGGTCCGCAAATCAATAAA |
| *MLS1* | GGAGCACCACTTGGAAGCTAAA | TGGAAAGCAGCAGGCAAA |
| *COX8* | TCAAAGTCAAAGGAAGAAAGACACC | GGCAACAAATGGAACAGCAA |
| *MRPL4* | GCTTGCTGCCGAAAACCATC | GAAGCGCTATAGGCCAGCTC |
| *MRPL3* | TAGAGAGGGGCTGGAGAAACC | AATGAGGAACCATAACCTTCACC |
| *MRPL17* | AACGACAGGATAACGGAAGCA | TTCATCAGAAAGATCGAAGTTAGGG |
| *MRPL39* | TGTGGTGAAACGGCATGTG | TTGGCTGTACGCAAGAAATCC |
| *MRPS35* | GGACTCAGGTGAGGCACTTTTC | GCTTCTTCGGGTGTTGTCTTC |
| *MRP13* | GGAACCATTACAACCGAGACC | TCCCTGTCCATTCTTTGAACC |
| *RSM25* | AAACGAAGCAGTATGGGGACA | AATGGCAGAAACTCCAAAGAGG |
| *RSM27* | TGGCAAGCTACTACGGCAATC | GCACCTTTACCACGACGTTTC |
| *MSY1* | ACAGAAACCGATGTGCTCTCA | ACAGAAACCGATGTGCTCTCA |
| *DIA4* | ACAGGGACGATAACACAGGAAA | CGGTGCTTGGGTCATAGAAA |
| *SLM5* | GGTGGGAGTTTAAGGGAAGATG | AGACCAAAGCCTCCGTGTG |
| *ISM1* | TGCTGGAAACGACGAGAGAA | CCAATGACGATATTTGGTTGGA |
| *MSF1* | CGGATTGATTCGTCAAGATGTG | GTCCAAACCCAAGCCAAAAG |
| *MSW1* | CGCCATGAGGCAGTAGCAAG | ATCCCATCGAGGCAAGAGTAGAAAG |
| *AIM36* | TGGTTAAAGGGTTGAAAAGACG | TCTTCAATGCTGGGTGATAAGG |
| *AIM11* | CAAAGGAATGAAGCCATGTCAG | AATGTCAAGTGCCCAACAAAAG |
| *AIM7* | CGAATTAGCCGAGATCCTACC | TCTTCCATTCCTGTGAAACGAC |
| *IBA57* | ACGAACCAAGAGAAAGCAAAGG | CAGATGAGAAGGCAGCAGAGAA |
| *FMC1* | AGCCGACTCTAAGTCATTACGTTCC | TCCTCGTATTCTCTTTGGTTGTCC |
| *MAM33* | AAGAACCGAATCAGGCGAAA | CATCCATTGCGTTGTTGTAAGG |
| *MCX1* | CAGTGGAAACAGCGAATCAAAG | TGAGGGACCAACGACAAGG |
