# Supplementary material for: Pbp1, the yeast ortholog of human Ataxin-2, functions in the cell growth on non-fermentable carbon sources
Source: PLoS One. 2021 May 13;16(5):e0251456. doi: 10.1371/journal.pone.0251456 (PMC8118320; doi:10.1371/journal.pone.0251456)
Supplement: S1 Fig — mRNA expression of genes in (A) MLS1, (B) COX8, (C) genes in group 3 (MRPL4, MRPL3, MRPL17, MRPL39), (D) genes in group 4 (MRPS35, MRP13, RSM25, RSM27), (E) genes in group 5 (MSY1, DIA4, SLM5, ISM1, MSF1, MSW1) (F) genes in group 6 (AIM33, AIM36, AIM11, AIM7) (G) genes in group 7 (IBA57, FMC1, AIM11, AIM7) in wild-type strain (WT) and pbp1Δ mutants growing in YPD and YPGL media. mRNA levels were quantified by qRT-PCR analysis, and the relative mRNA levels were calculated using 2-ΔΔCt method normalized to ACT1 reference gene. The data show mean ± SEM (n = 3) of fold change of mRNA level from wild-type cells at 4 h of culture in YPD. ns (not significant), *P < 0.05, **P < 0.01 as determined by Tukey’s test. (PPTX) [file pone.0251456.s005.pptx]

## Slide 1
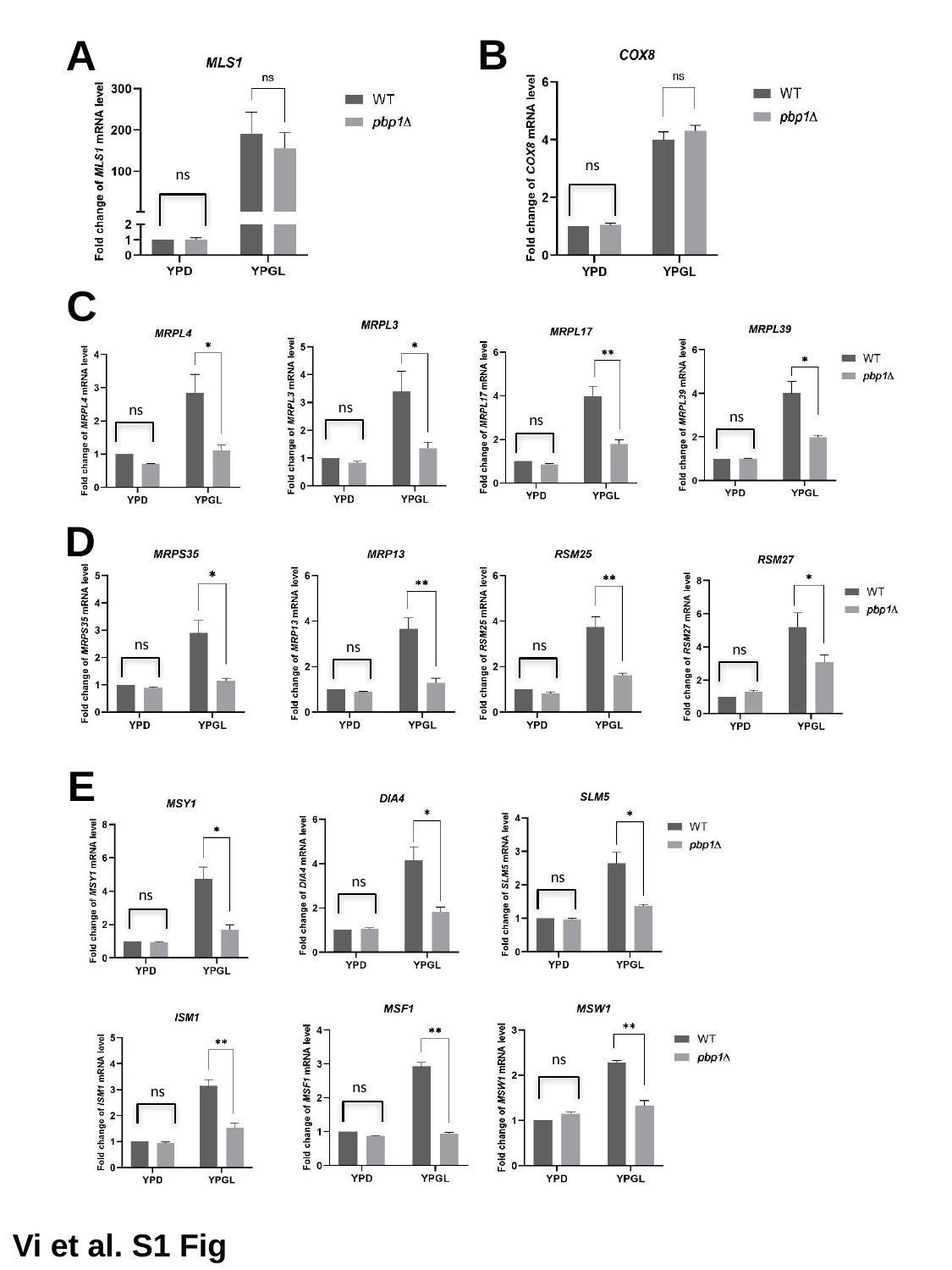

B
A
ns
ns
C
ns
ns
ns
ns
D
ns
ns
ns
ns
E
ns
ns
ns
ns
ns
ns
Vi et al. S1 Fig

## Slide 2
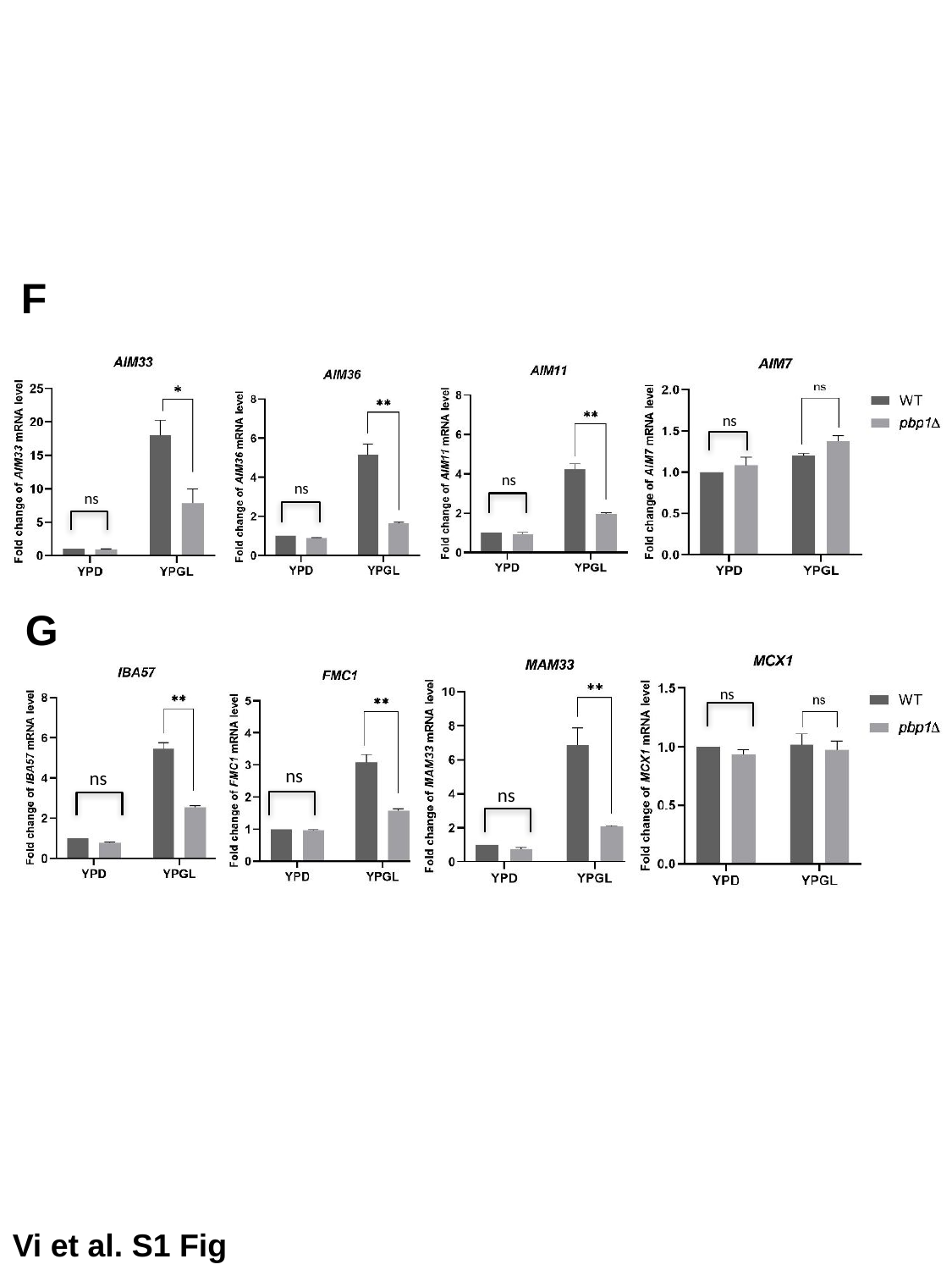

F
ns
ns
ns
ns
G
ns
ns
ns
ns
Vi et al. S1 Fig
