# Supplementary material for: Pbp1, the yeast ortholog of human Ataxin-2, functions in the cell growth on non-fermentable carbon sources
Source: PLoS One. 2021 May 13;16(5):e0251456. doi: 10.1371/journal.pone.0251456 (PMC8118320; doi:10.1371/journal.pone.0251456)
Supplement: S2 Fig — All the right panels display the mRNA levels of GFP gene driven by the promoter of (A) MRPL3, (B) MRPS35, (C) MSY1, (D) AIM33, and (E) IBA57 in wild-type strain (WT) and pbp1Δ mutant strain growing in YPD and YPGL media. The left panels display the mRNA level of the corresponding endogenous genes in the same strain as the right panels. After extracted, the total mRNA is used to measure mRNA level of both endogenous genes and GFP gene. mRNA levels were quantified by qRT-PCR analysis, and the relative mRNA levels were calculated using 2-ΔΔCt method normalized to ACT1 reference gene. The data show mean ± SEM (n = 3) of fold change of mRNA level from wild-type cells at 4 h of culture in YPD. ns (not significant), *P < 0.05, **P < 0.01 as determined by Tukey’s test. (PPTX) [file pone.0251456.s006.pptx]

## Slide 1
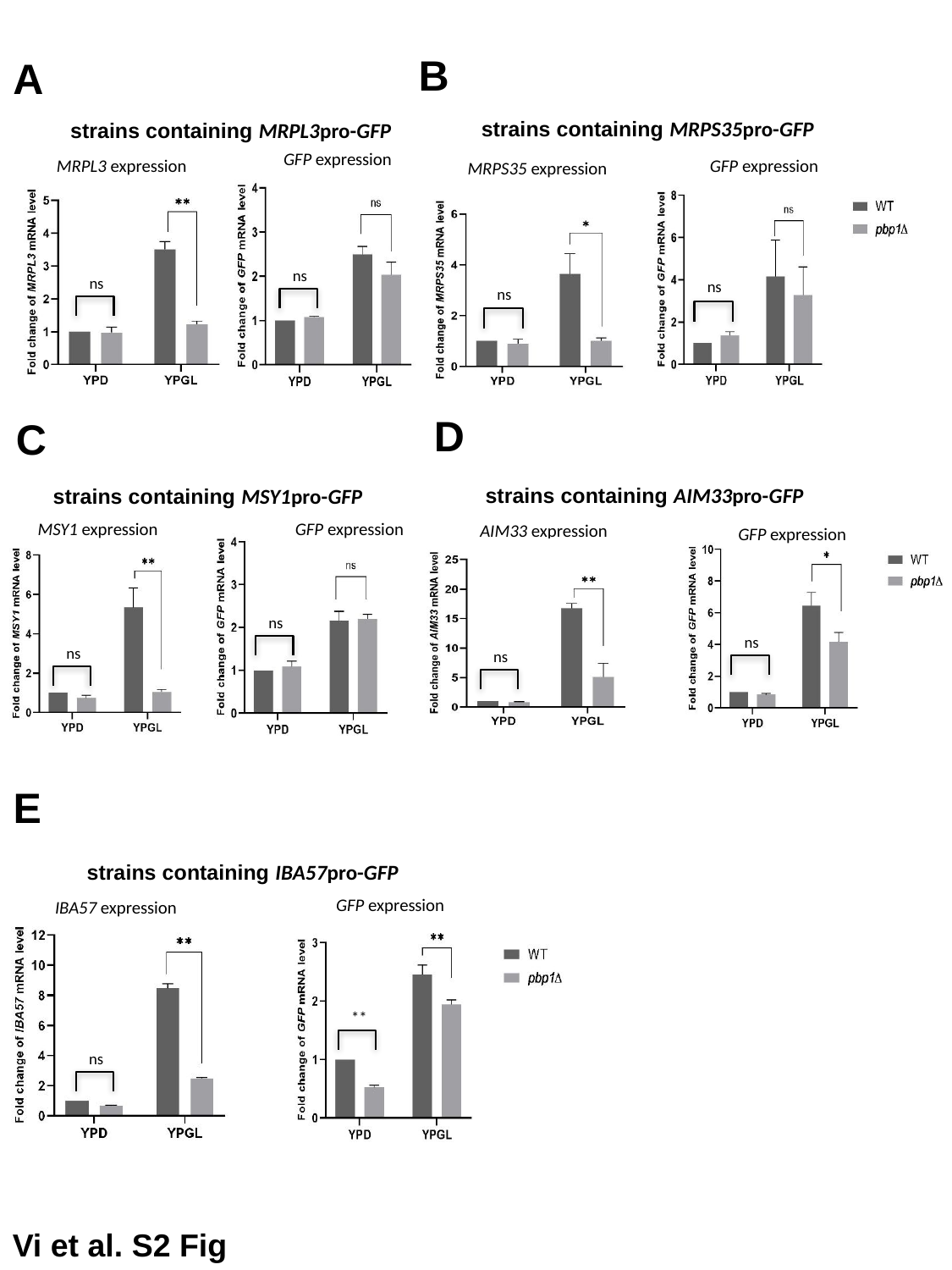

B
A
strains containing MRPS35pro-GFP
GFP expression
 MRPS35 expression
ns
ns
strains containing MRPL3pro-GFP
GFP expression
 MRPL3 expression
ns
ns
D
C
strains containing AIM33pro-GFP
ns
GFP expression
AIM33 expression
ns
strains containing MSY1pro-GFP
GFP expression
 MSY1 expression
ns
ns
E
strains containing IBA57pro-GFP
GFP expression
IBA57 expression
**
ns
Vi et al. S2 Fig
