# Supplementary figures and images for: Pbp1, the yeast ortholog of human Ataxin-2, functions in the cell growth on non-fermentable carbon sources
Source: PLoS One. 2021 May 13;16(5):e0251456. doi: 10.1371/journal.pone.0251456 (PMC8118320; doi:10.1371/journal.pone.0251456)

## Slide 1
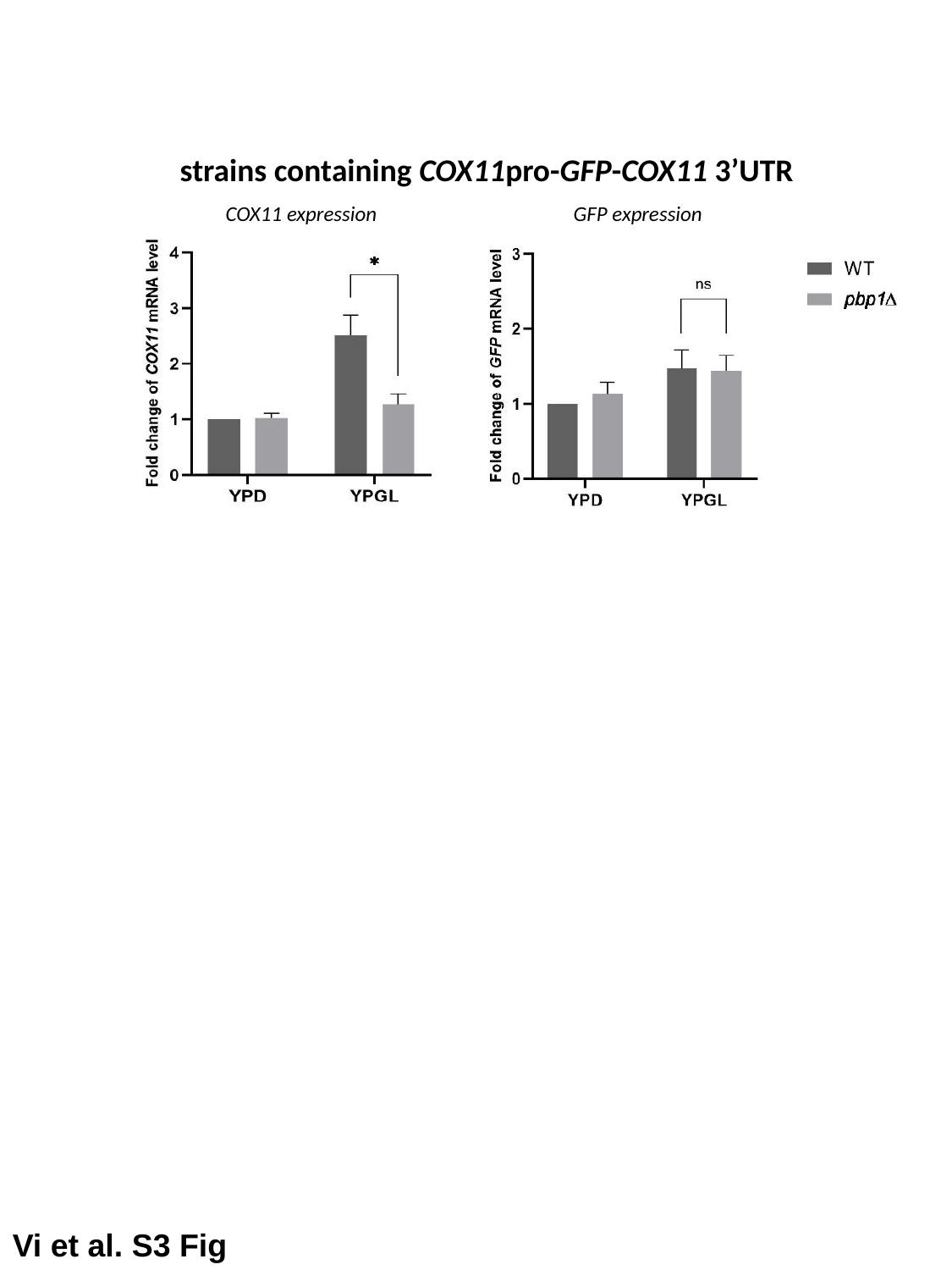

strains containing COX11pro-GFP-COX11 3’UTR
GFP expression
COX11 expression
Vi et al. S3 Fig

Supplement: S3 Fig — The level of GFP mRNA driven by COX11 promoter with ADH1 terminator (left panel) and with COX11 3’UTR (right panel). mRNA levels were quantified by qRT-PCR analysis, and the relative mRNA levels were calculated using 2-ΔΔCt method normalized to ACT1 reference gene. The data show mean ± SEM (n = 3) of fold change of mRNA level from wild-type cells at 4 h of culture in YPD. ns (not significant), *P < 0.05, **P < 0.01 as determined by Tukey’s test. (PPTX) [file pone.0251456.s007.pptx]

## Slide 1
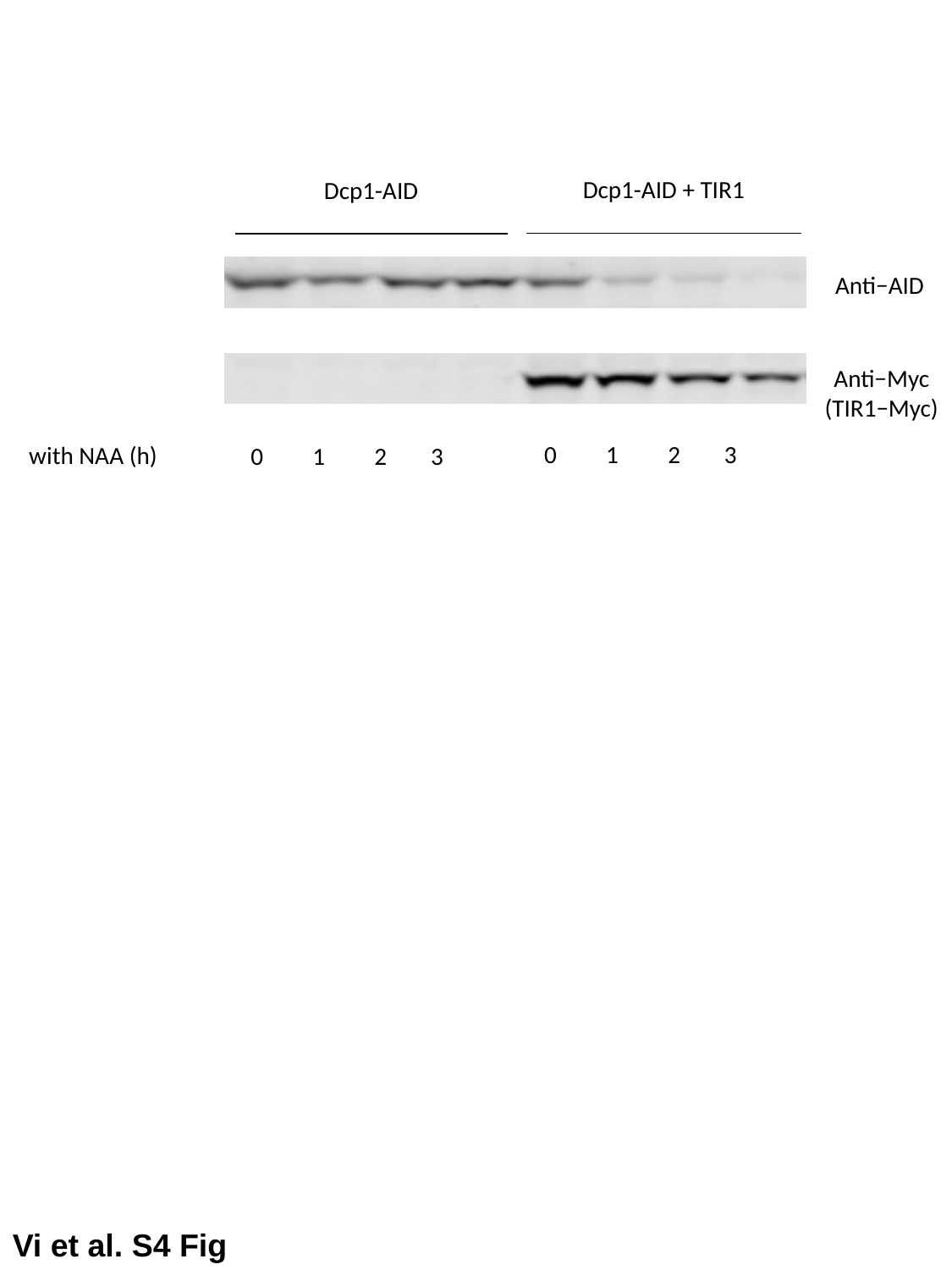

| Dcp1-AID + TIR1 |
| --- |
| Dcp1-AID |
| --- |
Anti−AID
Anti−Myc
(TIR1−Myc)
 0 1 2 3
with NAA (h)
 0 1 2 3
Vi et al. S4 Fig

Supplement: S4 Fig — The strain in which Dcp1 protein was fused with AID degron (DCP1-D), and pbp1Δ mutant with the same system (pbp1Δ DCP1-D) growing in YPGL media one hour after the addition of NAA. Dcp1-AID protein level was examined by Western blotting. (PPTX) [file pone.0251456.s008.pptx]
