# Supplementary material for: Pbp1, the yeast ortholog of human Ataxin-2, functions in the cell growth on non-fermentable carbon sources
Source: PLoS One. 2021 May 13;16(5):e0251456. doi: 10.1371/journal.pone.0251456 (PMC8118320; doi:10.1371/journal.pone.0251456)
Supplement: Attachment — Submitted filename: responses to the reviewers 20210420.docx [file pone.0251456.s009.docx]

**Our responses to the reviewers’ comments and changes in the revised manuscript**

PONE-D-21-05834

Tittle: Pbp1, the yeast ortholog of human Ataxin-2, functions in the cell growth on non-fermentable carbon sources

Authors: Dang Thi Tuong Vi, Shiori Fujii, Arvin Lapiz Valderrama, Ayaka Ito, Eri Matsuura, Ayaka Nishihata, Kaoru Irie, Yasuyuki Suda, Tomoaki Mizuno, Kenji Irie

**Reviewer 1**

**1.** Fig.1 and Fig.5, Time-dependent growth at different carbon sources should be shown, as shown in Figures 1D and 1F of Reference 26 (Yang et al. 2019). Please clearly indicate the number of repetitions. If for any reason a spot assay needs to be performed, serial dilutions of the medium containing yeast cells should be used, and the number of repeats of the experiments should be clearly indicated as well.

According to the reviewer’s comment, we have cultured WT strain and *pbp1∆* strain in YPD, YPRaff, and YPGL liquid media and generated a time series data of Optical Density (OD A_­600_) with 3 replicates. We showed this data in the revised manuscript (Fig. 1D). The growth curve of *pbp1∆* strain (doubling time 2.39 ± 0.08) is identical to that of WT strain (doubling time 2.57 ± 0.19) YPD liquid medium. Meanwhile, in YPGL liquid medium, the growth rate of *pbp1∆* strain (doubling time 8.25 ± 0.18) is remarkably lower than that of WT strain (3.87 ± 0.05). The result of growth curve in liquid media is consistent with tetrad assay (Fig. 1A) and spot assay (Fig. 1B) which was conducted in 3 replicates. We also conducted the complementation assay in which we reintroduced *PBP1* gene into *pbp1∆* strain. In this experiment, the reintroduction of *PBP1* gene recovered the growth deficiency of *pbp1∆* strain in selective synthetic medium containing glycerol lactate (-UraGL) (Fig. 1C). We added these figures and edited the sentences in the revised manuscript (page 10, lines 231-233). We also generated a time series data for WT, *pbp1∆, cat8∆, pbp1∆cat8∆* as Fig.6B and added text in the revised manuscript (page 15, lines 362-364)

**2**. Lines 122-132, Configurations of the solid medium (YPD, YPRaff, YPGL) used in Fig. 1 are missing.

We added the information about solid medium configurations in the revised manuscript (page 6, lines 130-137)

**3.** For experiments using *pbp1Δ*, it is necessary to test the effect of restoring *PBP1* expression.

We conducted the complementation assay in which we reintroduced *PBP1* gene into *pbp1∆* strain. We also introduced into WT and *pbp1∆* the backbone plasmid (YCplac33) to obtain control strains. We re-examined the expression of *PCK1, FBP1, ICL1, COX10*, *COX11, CYT2,* and *PBP1* in theses strains. Unlike in YPGL medium, the difference expression level of *PCK1*, *FBP,* and *ICL1* between wild-type and *pbp1∆* was not observed in synthetic medium (-UraGL). The decreased expressions of *COX10*, *COX11, CYT2,* and *PBP1* in the *pbp1∆* mutant in -UraGL medium were efficiently rescued by the *PBP1*-containing plasmid. We added this data as shown in Fig.3 and explained the result in the revised manuscript (page 12-13, lines 294-306).

**4.** Lines 130-131, describe the configurations of SC media.

We added the configurations of SC media on page 6, lines 130-137.

**5.** Line 142, “10 mL”

We corrected this in the revised manuscript (page 7, line 149)

**6.** For reagents and equipment used, indicate where the company is located (see recent research articles published in PLOS ONE, e.g. Hansen et al. doi:10.1371/journal.pone.0247375).

We added this information in the revised manuscript (Materials and Methods section)

**7.** Lines 165 and 179, Please cite Addgene plasmid number. (see <https://help.addgene.org/hc/en-us/articles/205432559-How-do-I-cite-a-plasmid-that-I-received-from-Addgene-in-future-publications->)

We cited Addgene plasmid numbers in revised manuscript (page 8, lines 174 and 187).

**Reviewer 2**

**1**. p.20, l.330-331

Based on the observation that *pbp1∆* and *cat8∆* show no synthetic defect in the induction of *PCK1* or *FBP1* (Fig.4), it was concluded that the regulation of Pbp1 may not be mediated by the transcriptional activator Cat8. Generally speaking, a lack of synthetic genetic defects is regarded as indicative that both factors act in the same pathway, so the discussion here is somewhat confusing. As a synthetic growth defect was observed indeed (Fig.5), the reviewer agrees that it is more likely that Pbp1 and Cat8 act in different pathways. However, the interpretation of the data shown Fig.4 should be handled more carefully.

In response to the reviewer’s comment, we re-interpreted the data with prudence in the revised manuscript (page 15, lines 364-366).

**2**.p.25,l.460-463

According to the Introduction section, glucose depletion induces TORC1 inactivation by Pbp1 through Pbp1 phosphorylation by Psk1 or methionine-rich region of Pbp1 (Pbp1 acts upstream to TORC1). On the other hand, discussion here sounds to presuppose a role of Pbp1 much closer to transcription (Pbp1 acts downstream of TORC1). Partly because the relationship between these two possibilities is not clear, the discussion here is confusing and should be revised.

We have modified the discussion to make the conclusion more explicit that Pbp1 is likely to act upstream of TORC1 (page 20, lines 482-488).

**3.**p.19,l.317
suppress catabolite

We modified this in the revised manuscript (page 14, lines 342)

**4**.p.26,l.491

transcripts level

We modified this in the revised manuscript (page 21, lines 515)
